# Identification of B cell antigens in solid cancer: initial insights and functional implications

**DOI:** 10.3389/fimmu.2025.1571570

**Published:** 2025-04-28

**Authors:** Jung-In Yang, Philip Moresco, Douglas Fearon, Min Yao

**Affiliations:** ^1^ Cancer Center, Cold Spring Harbor Laboratory, Cold Spring Harbor, NY, United States; ^2^ Graduate Program in Genetics, Stony Brook University, Stony Brook, NY, United States; ^3^ Medical Scientist Training Program, Stony Brook University Renaissance School of Medicine, Stony Brook University, Stony Brook, NY, United States; ^4^ Meyer Cancer Center, Weill Cornell Medicine, New York, NY, United States; ^5^ Sanders Tri-Institutional Therapeutics Discovery Institute, New York, NY, United States

**Keywords:** cancer antigen, B cell antigen, B cell function, single-cell sequencing, solid cancer

## Abstract

Cancer antigen discovery has mostly focused on T cell antigens, while antigens driving B cell responses have been largely overlooked despite representing another important branch of adaptive immune responses in cancer. Traditional B cell antigens in cancer have been studied using serological approaches analyzing polyclonal antibodies in serum. With recent technological advances in single-cell sequencing, a few studies have begun to investigate single B cell antigen specificity in the tumor microenvironment using immunoglobulin single-cell sequencing, recombinant monoclonal antibody production, cancer binding screening, and antigen identification. In this review, we highlight the initial insights into B cell directed cancer antigens and categorize them into cancer-associated viral antigens and non-viral antigens, with the latter featuring autoantigens. We will further discuss the functions of B cells in cancer in the context of their antigen specificity, and categorize their functions into antibody effector function, T cell activation, and B cell secretion. Lastly, we will provide perspectives on the challenges and opportunities in the identification of new B cell cancer antigens and highlight their translational potential.

## Introduction

Within the tumor microenvironment (TME), a complex interplay among cancer cells, stromal cells, and immune cells occurs to establish a niche that will either facilitate cancer outgrowth or immune-mediated tumor control. Cancer antigens that elicit adaptive T and B cell responses provide important insights into underlying cancer immunity and can be utilized to augment cancer immunotherapy. The field of cancer immunology has mainly centered upon studying T cell- targeted cancer antigens, largely neglecting cancer antigens activating the B cell response. Recently, increased presence of tumor infiltrating B cells and tertiary lymphoid structure (TLS) have been associated with favorable immunotherapy responses in various cancers (1). However, animal studies report conflicting roles of B cells in different cancer models, including both pro- and anti-tumorigenic niches (2, 3). The B cell mediated anti-cancer effect is suggested through antigen presentation to T cells and antibody mediated effector function. On the other hand, B cell mediated pro-tumor effects are proposed to be related to their secretion of immunosuppressive cytokines. Some of the discrepancies in B cell functionality in cancer may result from the use of different tumor models (2). In addition, the lack of in-depth understanding of B cell antigens in cancer also contributes to the inconsistent interpretation of B cell functions. Identification of B cell antigens in cancer will provide important insights in B cell function, and the identified cognate antibody/antigen pairs could offer potential therapeutic opportunities.

**Figure 1:**
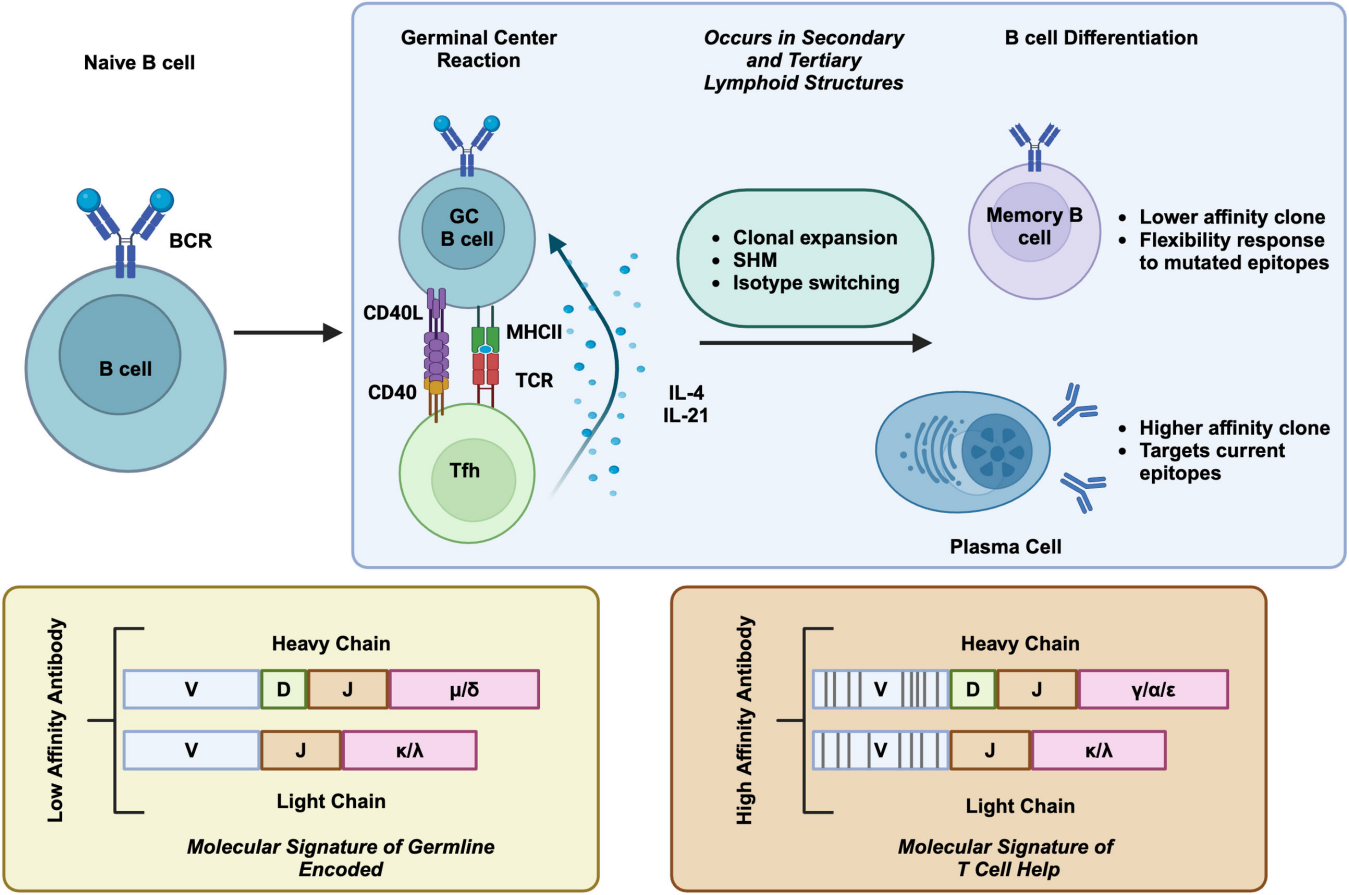
T cell dependent B cell activation. When naive B cells interact with their cognate antigen, they undergo a proliferation and differentiation process that is assisted by Tfh cells. Tfh interaction helps induce clonal proliferation, variable region SHM, and isotype switching. Selected GC B cells differentiate into memory B cells and plasma cells. The molecular signature of T cell dependent B cell activation are featured in below, containing SHM and isotype switch, in comparison to germline encoded naive B cells. Created in BioRender. Moresco, P. (2025) https://BioRender.com/fc1sifv.

In adult mammals, B cells develop from the bone marrow through lineage-specific transcription factors, which coincide with homologous recombination of V, D, and J segments of immunoglobulin genes (4). The recombined immunoglobulin is expressed as membrane-bound IgM and complexes with CD79A and CD79B to form the B cell receptor (BCR) (5), which marks the immature B cell. In the bone marrow, B cells undergo negative selection to remove self-reactive BCRs, a process termed central tolerance. During this process, high-affinity self-reactive B cell clones are eliminated by clonal deletion, or anergy, or via the second light chain rearrangement to edit BCR (6). However, this process is imperfect as around 20% of naïve B cells still contain low levels of self-reactivity (7) and are further controlled by peripheral tolerance mechanisms (8). Immature B cells leave the bone marrow and enter the spleen to mature into naïve B cells (9). Naïve B cells are activated when they encounter their cognate antigen in secondary lymphoid organs, such as lymph nodes. B cell activation requires both BCR binding to antigen and co-stimulation by additional cytokines (10). B cell activation by protein antigen mostly requires CD4+ follicular helper T (Tfh) cells, through their coordinated interaction, called the germinal center reaction. In this process, B cells will internalize their antigen via the BCR, present the antigen complexed on MHC-II to Tfh cells, which recognize the same antigen through their T cell receptor (TCR) (10). Tfh cells further drive B cell activation via CD40L/CD40 signaling and through the secretion of stimulating cytokines such as IL-4 and IL-21 (11). These signaling cascades drive B cell clonal expansion, isotype switching (IgM to IgG/IgA/IgE), and V region somatic hypermutation (SHM) to achieve affinity maturation (10). The germinal center reaction marks immunoglobulin genes with intrinsic genetic features which distinguish antigen-experienced B cells from naïve B cells (**Figure 1**). After this process, B cells will further differentiate into antibody-secreting plasmablasts (PB), plasma cells (PC), or memory B cells (10).

In cancers, B cell subtypes include the classic naïve, activated, germinal center, and memory B cells, and antibody-secreting PBs and PCs (2). In addition, other B cell subtypes in cancer include regulatory B cells (Bregs) and atypical B cells (ABCs). Bregs are characterized by their expression of immunosuppressive cytokines, such as IL-10, IL-33, and TGFβ, and have been primarily studied in mouse tumor models (2). ABCs have been described using single-cell RNA profiling in cancer, generally defined as IgD^-^, CD27^-^, FCRL4^+^, CD11c^+^, and TBX21^+^ memory B cells (12–15), and are similar to ABCs reported in autoimmune disease and aging (2). However, their functionality in cancer is not well-characterized. A more thorough description of these cell types is reviewed elsewhere (2).

**Figure 2:**
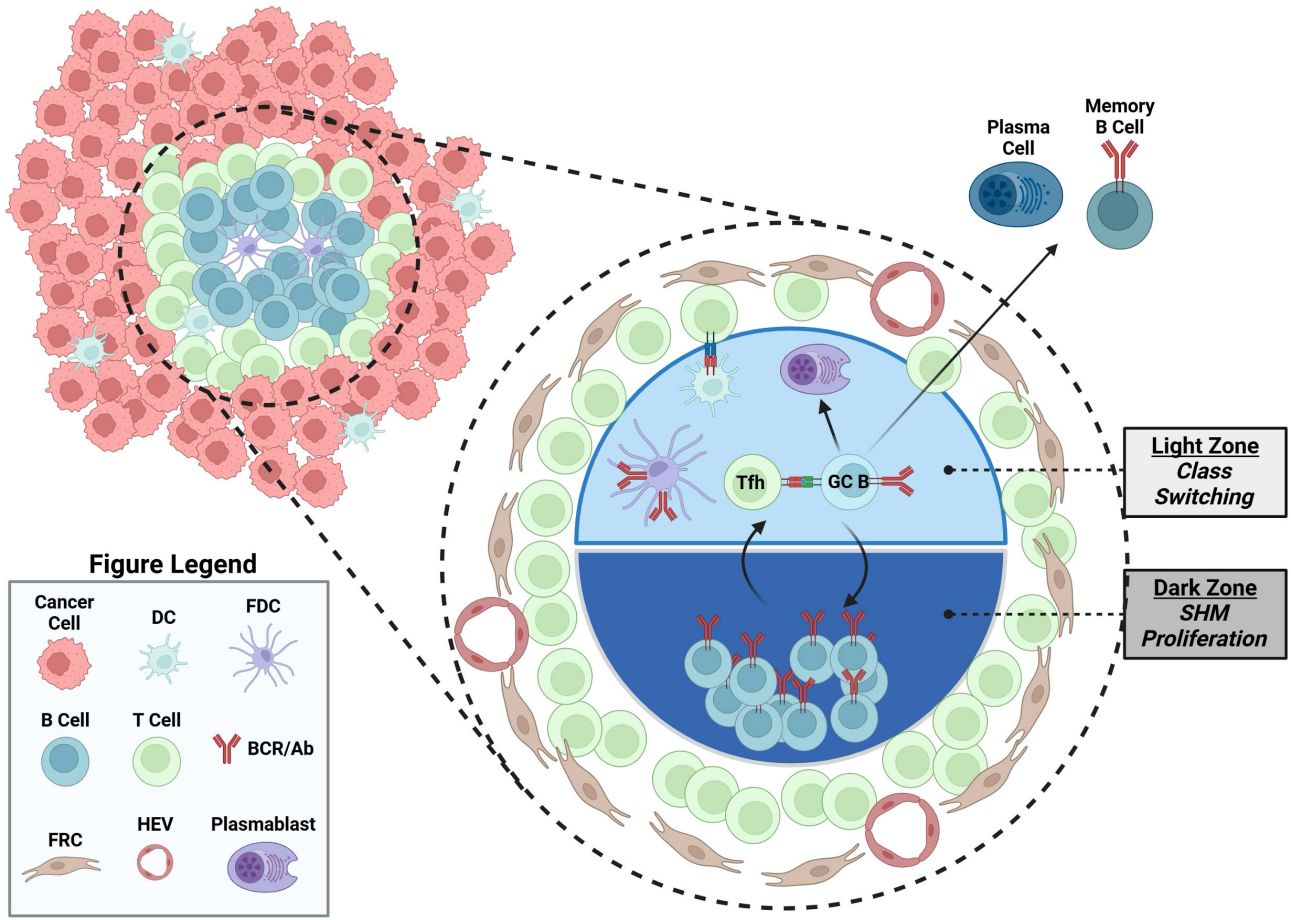
An illustrated TLS containing a germinal center. TLS located in cancer contains germinal center, where B cells undergo local antigen driven T cell dependent germinal center activation, including somatic hypermutation and proliferation in the dark zone, and Tfh cell selection and class switch in the light zone. Other cell types in TLS are also illustrated, including dendritic cells (DC), follicular dendritic cells (FDC), follicular reticular cells (FRC), plasma cells, memory B cells and high endothelial venules (HEV). Created in BioRender. Moresco, P. (2025) https://BioRender.com/qilbcv4.

The TLS is a non-encapsulated ectopic lymphocyte aggregate formed under chronic inflammation conditions, including autoimmune disease, transplantation rejection, and cancers (1). Similar to encapsulated secondary lymph organs, the TLS primarily contains B and T cells, organized into lymphoid follicles, surrounded by dendritic cells, macrophagess, PCs and stromal follicle reticular cells, and high endothelial venules (**Figure 2**). In general, the presence of TLS and B cells is associated with favorable outcomes and response to checkpoint blockade therapy in various human cancers (16–20), such as melanoma, sarcoma, breast cancer, and renal cell carcinoma. This topic has been reviewed extensively previously (1). The features of PCs are also strongly associated with a favorable outcome in some cancer types (21–23). Despite these well-established correlations, the formation and function of the TLS in cancer are still not well understood (1). It is postulated that under the chronic inflammatory state seen in cancer, there is production of TLS inducing signals, including lymphotoxin and TNF, and chemokine production such as CCL19, CCL21 and CXCL13, which help recruit T and B cells (1). B cells potentially contributed to the formation of TLS through their expression of lymphotoxin-α1β2, exemplified in a murine melanoma model (24). The unencapsulated TLS structure facilitates the sampling of local tumor derived antigens to augment B and T cells responses. In addition, the TLS may also provide survival signals for aggregated immune cells in cancer, such as BAFF and APRIL for B cells and PCs (25). In support of this, the mature TLS exhibits features of germinal center reactions, and BCR sequencing from TLS has revealed classic B cell activation markers of clonal expansion and SHM (1). Those data support the idea of B cells in TLS undergoing local antigen driven activation, providing a rationale for sampling B cells within TME to identify B cell directed cancer antigens (1).

The antigens that drive B cell activation in cancer are largely unexplored. Historically, B cell antigens in cancer are studied using polyclonal antibodies in peripheral blood (the serologic approach). However, the polyclonal nature of the antibodies studied in this method limits antigen identification. In addition, the antibody response in peripheral blood may not fully reflect the local B cell response in the TME. Recently, the advancements of single-cell sequencing technology enabled the study of B cell antigen reactivity in the TME at a single-cell level (26). Single cell BCR sequencing facilitates the production of tumor sourced recombinant monoclonal antibodies, which allow for subsequent cancer antigen screening and antigen identification. A few recent studies have investigated individual B cell antigen specificity in the TME using this approach. In this review, we summarize recent advancements in B cell antigen identification and the B cell response in solid cancers, providing insights for potential therapeutics in cancer immunotherapy.

## B cell antigens in human solid cancer

The identification of B cell directed cancer antigens can be broadly broken down to include either serological or single B cell approaches. Traditionally, serological sampling of antibodies was the most common method employed, as collecting antibodies from peripheral blood was a convenient method of sequentially monitoring antibody responses during treatment or cancer progression. Furthermore, the identification of the serum antibody reactivity was facilitated with assays such as candidate antigen screening (27–29), SEREX (30), phage display (31, 32) and protein array (33). Candidate antigen and protein array approaches use a selected list of candidate antigens or an array of candidate antigens to probe serum derived antibody binding. SEREX identifies potential cancer antigens by analyzing serologic binding to cDNA expression library derived from cancer (30). Similarly, phage display identifies potential cancer antigens by analyzing serologic binding to a phage library expressing synthetic or patient derived peptides (32). In general, serological profiling of antibodies identified numerous autoantibodies that bound to self-antigens expressed in both cancer cells and normal tissue, including the nuclear antigen p53 (27), cancer testis antigen NY-ESO-1 (28), and glycosylated surface protein MUC-1 (34). Prior reviews have summarized serologic studies on cancer antibody reactivity (35–37) and thus will not be of focus here. With this said, an important caveat in assaying a serologic response to cancer is the fact that the peripheral blood antibodies may be resulted from immunological responses unrelated to the oncological process. In addition, the polyclonal mixture of antibodies in blood also makes downstream antigen identification challenging.

**Figure 3:**
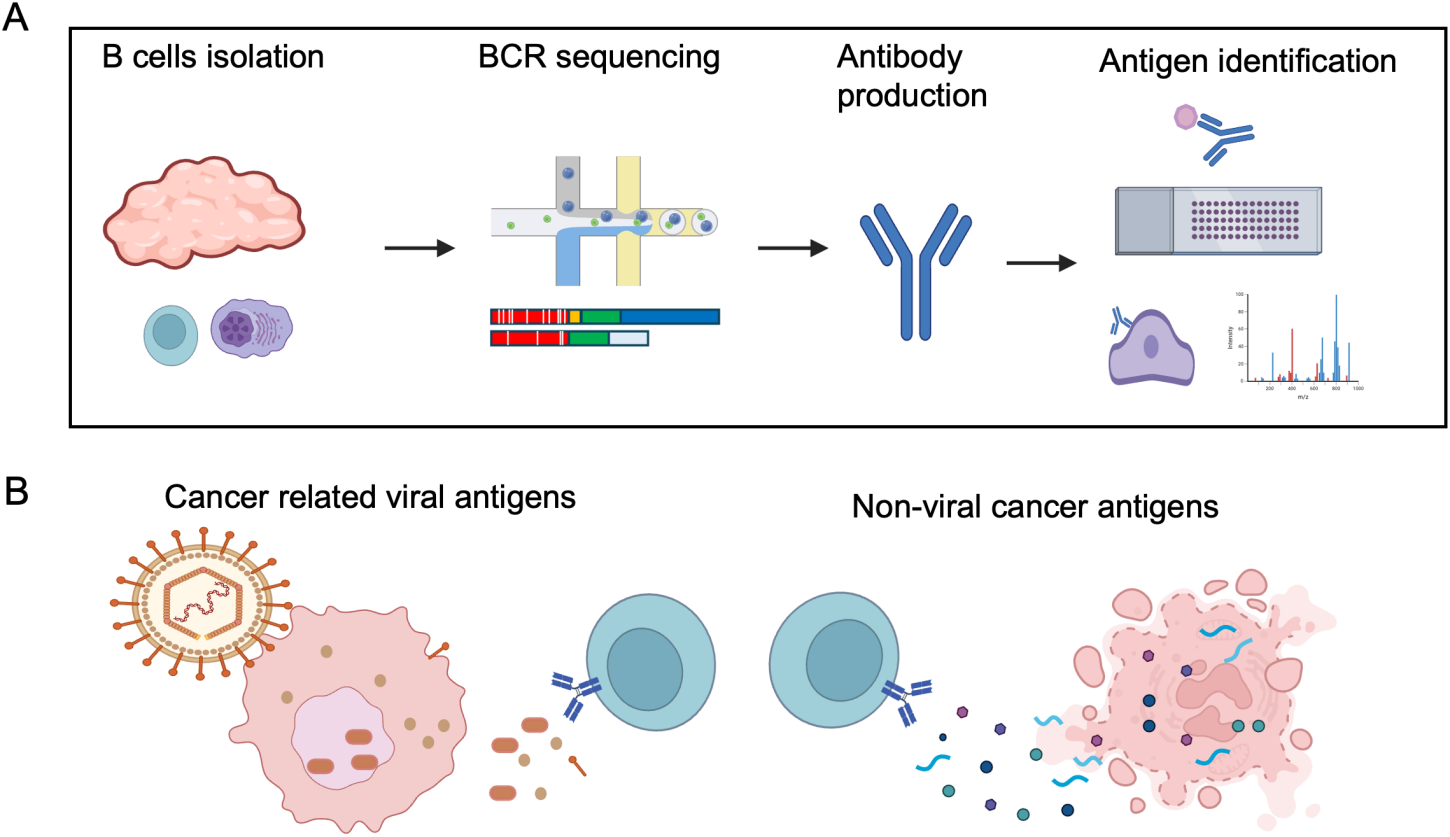
B cell directed cancer antigen identification using single-cell approach. **(A)** Example of B cell antigen identification by single B cell method, consisting of: 1) Isolation of B cells and plasma cells from cancer samples; 2) Single-cell BCR sequencing; 3) Production of recombinant antibodies; 4) Screening antibodies binding to candidate targets, protein array or cancer samples, and followed by antigen identification by immunoprecipitation and mass spectrometry. **(B)** Illustration of cancer related viral antigens and non-viral cancer antigens, both dominated by intracellular antigens, likely released during cell death. Most of the identified non-viral cancer antigens are also self-antigens. Created in BioRender. Moresco, P. (2025) https://BioRender.com/not3uny.

Identifying the immunoglobulins of B cells and PCs within the TME is an appealing method to identify B cell targeted cancer antigens. Single-cell BCR sequencing allows direct sampling of B cells and PCs within the TME, facilitating the identification of cells undergoing clonal expansion, isotype switching, and somatic hypermutation in response to tumor antigens. Subsequent recombinant monoclonal antibodies produced from BCR sequencing can then be used to identify the antigens being targeted by the intratumoral B cell response via candidate antigen screening, human protein arrays, and mass spectrometry-coupled immunoprecipitation (**Figure 3**). We summarize a few recent studies using these approaches, and their identified antigens in the following section (**Table 1**).

**Table 1:** Summary of identified B cell antigens in human solid cancers.

Cancer type	Type of antigen	Identified antigens	Antigen location	Antigen function	References
Head and neck cancer	Viral	E2 (HPV)	Intracellular	Viral transcription regulation	(38)
		E6 (HPV)	Intracellular	Viral oncogene, p53 degradation	(34)
		E7 (HPV)	Intracellular	Viral oncogene, Rb degradation	(34)
Nasopharyngeal carcinoma	Viral	EBNA1 (EBV)	Intracellular	Promote viral genome stability and cell survival	(37)
		VCA (EBV)	Intracellular	Viral nucleocapsid protein	(37)
		BNLF2b (EBV)	Unknown	Function unknown	(37)
Lung cancer	Viral	HERV-K envelope glycoprotein (ERV)	Cell surface	Viral envelope glycoprotein	(44)
Breast medullary carcinoma	Non-viral	β-actin	Intracellular	Actin cytoskeleton	(51)
Ovarian carcinoma	Non-viral	MMP14	Cell surface	Matrix metalloproteinase	(51)
		BDNF	Secreted	Neuronal growth factor	(52)
		TSPAN7	Cell surface	Synaptic development and function	(48)
Melanoma	Non-viral	TUBB1, TUBB2A, TUBB4A, TUBB4B	Intracellular	Tubulin cytoskeleton	(56)
Pancreatic ductal adenocarcinoma	Non-viral	F-actin	Intracellular	Filament actin, actin cytoskeleton	(57)
		RUVBL2	Intracellular	Transcriptional regulation	(57)
		HSPD1	Intracellular	Mitochondrial heat shock protein	(57)

## Cancer-related viral antigens

Viral-associated solid tumors are expected to induce B cell responses to foreign viral antigens. In human papillomavirus (HPV) positive head and neck cancers (HNSCC), Wieland et al. reported that plasma IgG titers to three candidate HPV antigens, E2, E6, and E7, were significantly higher in HPV positive HNSCC than other HNSCC, with E2 being the dominant antigen (38). All three HPV antigens are intracellular proteins, with E2 being a DNA-binding protein, and E6 and E7 being viral oncogenes that inhibit p53 and Rb, respectively (39). Wieland et al. identified that HPV-specific memory B cells and PCs were enriched in the TME but not in peripheral blood by an ELISPOT assay, an example of a local antigen specific response. The authors further isolated E2-specific monoclonal antibodies by antigen-specific B cell sorting, and these antibodies demonstrated a high degree of SHM through BCR sequencing (38).

Nasopharyngeal carcinoma (NPC) is commonly associated with Epstein-Barr Virus (EBV) infection (40). Serologic IgG or IgA response to EBV proteins is prevalent in patients with NPC, and about 20 EBV antigens have been reported to induce serologic responses (41). Among these, the dominant antigens are intracellular proteins, including the nuclear antigen EBNA1, viral nucleocapsid protein VCA, and the recently identified BNLF2b whose function is mostly unknown (41). EBV viral DNA detection (42) and the measurement of serologic antibodies to these EBV proteins (43) have been used clinically as screening tools for the early detection of NPC. Though no studies have used a single-B cell approach to identify EBV reactive B cells in the NPC TME, B cells and PCs targeting EBV antigens are expected in this cancer type.

Ng et al. reported that endogenous retroviruses (ERVs) induce an anti-tumor B cell response in a mouse model of lung cancer (44). Furthermore, the authors detected elevated ERV mRNA expression in human lung cancer samples relative to healthy controls. Serological antibody responses to those human ERVs (HERV-K) were detected in approximately 20% of lung cancer patients. However, the antibody response did not correlate with ERV mRNA expression. Thus, the prevalence of ERV-induced B cell response in human cancer needs further investigation.

In addition, viral antigens are also expected to induce a B cell response in other virus associated cancers, such as HPV infected cervical, endometrial, and ovarian cancer (45, 46), hepatitis B or C virus infected liver cancer (47, 48), and cytomegalovirus infected cancers (46, 49, 50). Further studies to investigate virus induced B cell responses at the single-B cell level will be helpful to gain further insights.

## Non-viral cancer antigens

In non-viral associated cancers, a few studies have begun to examine the antigen specificity of intratumor B cells using single B cell approaches. These studies include reports in melanoma, breast, ovarian, and pancreatic cancer, revealing that these B cell responses commonly target autoantigens.

Two studies have identified B cells targeting secreted or cell membrane self-antigens in cancer. In high-grade serous ovarian carcinoma (HGSOC), Mazor et al. showed abundant IgG coating on the surface of cancer cells (51). Single cell sequencing of PCs in the TME showed significant SHM of their immunoglobulin genes, reflecting affinity maturation likely via a tumor-antigen-driven antibody selection. The authors screened recombinant monoclonal antibodies against selected candidate proteins of the extracellular matrix and identified antibodies frequently bound to matrix metalloproteinase-14 (MMP14), with some cross-reactivity to other MMP family members. The authors further identified that some MMP14 autoreactive antibodies had undergone SHM, while others came from germline-encoded VDJ sequences, indicating germline dependent and independent autoreactivity. However, those identified antibodies bound to MMP14 with a relatively low affinity (EC50 > 100 nM), despite containing a high degree of SHM, raising the question of whether they may recognize additional unidentified cancer antigens.

In another study of HGSOC, Biswas et al. characterized PCs from dissociated tumor samples and discovered the predominant antibody isotype to be class-switched IgA (52). Tumor cells from these patient samples were also coated with IgA. The authors generated immortalized B cells from patient tumor tissue and screened secreted antibodies for their ability to detect antigens in human protein arrays, which led to the identification of antibodies recognizing selfantigens such as TSPAN7 (a cell membrane protein) and BDNF (a secreted growth factor). TSPAN7 is overexpressed in many epithelial cancers (53), and high serum BDNF levels are associated with HGSOC recurrence (54). Recombinant TSPAN7 and BDNF IgA antibodies are confirmed to bind tumor cells and recombinant proteins, and control mouse tumor growth. The effector mechanism of these antibodies will be discussed in a later section.

Besides the few examples of antibodies targeting extracellular proteins, antibodies targeting intracellular proteins are most commonly observed in cancer. An early study of medullary carcinoma of the breast (MCB) examined intratumoral B cell antigens using phage display (55). The authors found that MCB with B cell infiltration was associated with a better prognosis. They generated a phage display library using V(D)J cDNA isolated from MCB tissue and screened it binding to MCB histological sections. Recombinant antibodies from enriched phages were recombinantly synthesized and used for immunofluorescent staining, western blot analysis, ELISA, and mass spectrometry to facilitate antigen identification. The authors identified β-actin, a ubiquitous intercellular cytoskeleton protein, as the major B cell targeted cancer antigen.

Melanoma, which is known to have a significant anti-tumor T-cell response, also exhibited a similar pattern of B-cell reactivity to autoantigens (56). Differentiated B-cells were enriched in the tumor niche in melanoma, which showed evidence of clonal expansion, class switch recombination, and SHM. Crescioli et al. produced 12 recombinant antibodies from single-cell sorted memory B-cells and used the antibodies to identify target antigens from melanoma and skin tissue lysates (56). Antibody immunoprecipitation and mass spectrometry identified candidate self-antigens, including the tubulin cytoskeletal protein members (TUBB1, TUBB2A, TUBB4A, TUBB4B) and others. However, these antigen candidates were not further validated using recombinant protein or other additional assays.

In our own study in human pancreatic ductal adenocarcinoma (PDAC), which is generally considered an immunologically cold cancer, we identified a robust B cell response to intracellular self-antigens (57). We observed an active T and B cell response using single cell sequencing in seven microsatellite stable PDAC specimens, which were accompanied by histological features of TLSs. BCR single cell sequencing of PCs isolated from the same tumor samples revealed a robust class switching to IgG and IgA and a high degree of SHM. 41 antibodies representing the most dominant clones were recombinantly produced and screened for their ability to bind to PDAC samples using immunofluorescence staining. 21 of the 41 antibodies showed positive binding to antigens expressed in human PDAC cells, all of which were intracellular and also expressed in non-tumor fibroblast cells. Using immunoprecipitation and mass spectrometry, we were able to identify three target antigens: the intracellular self-proteins filamentous actin (cytoskeleton protein), RUVBL2 (nucleus protein), and HSPD1 (mitochondrial protein). Our study showed the dominant antibody response in PDAC is specific to intracellular self-antigens.

## The function of B cells in cancer

**Figure 4:**
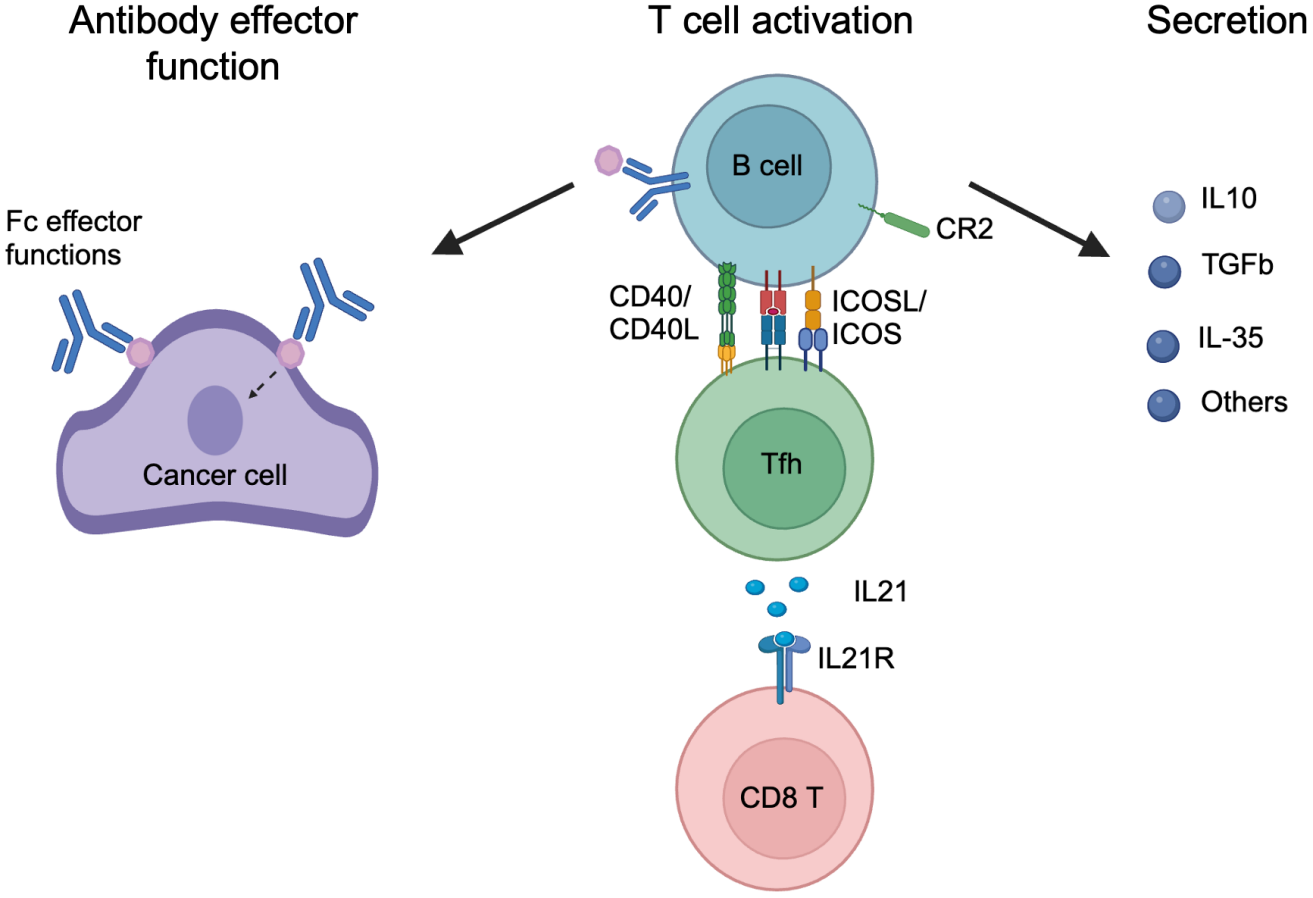
The mechanisms of B cell function in cancer. B cell functions in cancer are categorized: 1) Antibody effector function, in which secreted antibodies bind to antigen targets in cancer and mediated effector function through Fc mediated effector function or antigen binding signaling; 2) T cell activation, in which activated B cells interact with Tfh cells through CD40/CD40L, MHCII antigen presentation and ICOSL/ICOS, and leading to Tfh activation, and secretion of IL-21 that activates CD8 T cells through IL21R; 3) Secretion, activated B or PC cells secret a plethora of cytokine and metabolites to regular the tumor, including IL-10, TGFb, IL-35, and others. Created in BioRender. Moresco, P. (2025) https://BioRender.com/w62qayt.

How B cells affect long-term cancer outcomes is inconclusive, as both pro- and anti-tumorigenic functions have been attributed to B cells in different studies, and have been extensively discussed in previous reviews (2, 3). We think the lack of an in-depth understanding of B cell reactivity in cancer also contributes to the inconsistent interpretation of B cell functions in cancer. In this section, we summarize the function of B cells in cancer into three categories, with a focus on antigen recognition, and further discuss them below: 1) Antibody-mediated effector function; 2) Activation of T cells; 3) Secretion of cytokines and other modulators (**Figure 4**).

### Antibody-mediated effector function

B cell derived antibodies can impact cancer progression by directly binding target antigens or indirectly through binding to Fc receptors or fixing complement. These processes are termed: 1) antibody-dependent cellular cytotoxicity (ADCC), in which antibodies recognize surface antigens and stimulate natural killer (NK) cells via Fc receptor to lyse target cells; 2) complement-dependent cytotoxicity (CDC), in which IgG or IgM binds antigens on target cells leading to activation of the complement cascade and subsequent lysis of target cells; 3) antibody-dependent cellular phagocytosis (ADCP), in which antibodies opsonize target cells to facilitate phagocytosis; 4) finally, antibody binding to surface antigens can potentially stimulate or block their downstream signaling. While the occurrence of these processes is speculated in cancer, the direct evidences are limited to mouse studies and discussed below.

Examples of ADCC have been shown in mouse tumor models. Using a transplanted murine lung cancer model expressing the murine leukemia virus (MLV) (44), Ng et al. showed these tumors induced a robust anti-MLV B cell response and responded to checkpoint blockade therapy. Administering an antibody targeting the MLV surface glycoprotein enhanced tumor control in a NK cell-dependent manner, suggesting an ADCC-mediated antibody effector function. Similarly, Hollern et al. reported that engineered high-mutational load mouse breast cancer models induced T and B cell activation, and B cells were required for checkpoint blockade therapy. B cells were hypothesized to exert their tumor control through ADCC because tumors formed in mice deficient in antibody secretion, or the Fc receptor CD16, lacked tumor control, though the antigen specificity was not determined.

Conversely, Gu et al. showed that antibody-mediated antigen recognition promotes mouse mammary lymph node metastasis (58). Here, the authors found that the murine 4T1 mammary tumor model induced an IgG response targeting glycosylated HspA4 located on the cancer cell surface. IgG binding to HspA4 leads to activation of NF-kB signaling and lymph node metastasis. It is unknown whether antibody ADCC effector function may also be present in this tumor model, given the cell surface target of HspA4.

Biswas et al. reported that IgA antibodies control HGSOC growth (52). As discussed in Part I, the authors identified IgA responses to BDNF and TSPAN7 autoantigens in ovarian cancer and found that recombinant BDNF and TSPAN7 IgA antibodies reduce xenograft tumor growth. The BDNF antibody likely blocks BDNF signaling (though not directly proven), as the same antibody lacking the Fc domain still retained tumor control ability. Conversely, the TSPAN7 IgA antibody mediated tumor control requires the Fc region and is independent of NK cells, and promotes ADCP *in vitro*. The polymeric immunoglobulin receptor (pIgR) mediated IgA transcytosis also contributed to the tumor control, as pIgR knockout reduced BDNF and TSPAN7 IgA mediated tumor control.

Crawford et al. reported a robust IgE antibody response in a mouse skin tumor model induced by the carcinogen DMBA (59). Here, IgE promoted tumor control as knockout of IgE, or IgE receptor FcER1, resulted in increased tumor growth. In this study, IgE antigen specificity was not defined, but serum profiling indicated the IgE response potentially targeted self-proteins in the nucleus and cytoplasm.

### Activation of T cells

B cells can directly activate CD4+ T cells, primarily Tfh cells, by antigen presentation through MHC-II (11). The reciprocal signaling between T and B cells is critical for B cell activation, Tfh differentiation, TLS formation, and ultimately CD8 T cell function. Murine tumor models have revealed detailed mechanisms of B cell mediated CD4 and CD8 T cell activation.

Cui et al. used a mouse lung cancer cell line co-expressing the B cell model antigen Hen-egg lysozyme, and CD4 T cell antigen GP66, to study the reciprocal signaling between B and T cells (60). Compared with a mouse cell line expressing the CD4 T cell antigen alone, the addition of the B cell antigen resulted in the induction of a GC reaction and antigen specific Tfh cell generation. Both B and Tfh cells were critical for tumor control, as implanting tumors in mice lacking B cells (uMT mice), functional MHC-II (*CDIIA* knockout mice), and Tfh cells (*Bcl6* knockout in the CD4 lineage) resulted in increased tumor growth. The adoptive transfer of antigen-specific B or CD4 T cells was able to rescue the Tfh cell deficient mouse phenotype. Mechanistically, GC B cells promoted IL-21 expression in Tfh cells, which in turn promoted CD8 effector differentiation, as IL-21 blockade, *Il21* knockout, and *ll21r* knockout mice had enhanced tumor growth. This study provides an elegant and detailed mechanistic study on B and Tfh cell function in the activation of CD8 T cells in tumor control. A similar conclusion comes from a separate study using an engineered high-mutational load murine mammary tumor model (61), in which B cells, CD4 cells, and CD8 cells contributed to checkpoint blockade mediated tumor control. Antibody blockage of IL-21 had a similar effect as CD4 depletion, suggesting a potential common mechanism of action of Tfh cell activation by B cells expressing IL-21 to induce cytotoxic CD8 T cells. Though in the latter study, the B cell antigen specificity was not addressed.

Lu et al. reported ICOSL+, CR2+ B cells promoted tumor control during chemotherapy in mouse breast cancer models (62). Depletion of B cells, or conditional knockout ICOSL in B cells, reduced this tumor response to doxorubicin treatment and correlated with reduced CD4 and CD8 effector cells and increased Treg cells. The authors reported that complement signaling through CR2 is required for upregulation of ICOSL in B cells, demonstrated by *C3* (upstream complement activator) knockout mice, or conditional knockout of *Cr2* in B cells. Though the authors did not examine Tfh and CD8 T cell functions in this tumor model, ICOSL is known to be induced in GC-B cells during T-B interaction (63), and can activate Tfh cells through ICOS and potentially further promote CD8 T cells. While this study did not investigate the B cell antigen specificity, it is likely reactive to cancer antigens induced by chemotherapy.

### B cell secreted cytokines and other immunomodulators

B cell activation also leads to the production of cytokines and other immunomodulatory molecules. A few recent studies have highlighted examples of how these B cell derived molecules can regulate the tumor immune response.

B cells are reported to secrete cytokines to regulate tumor growth. Wang et al. showed that Tgfβ1 secreted from Leucine-tRNA-synthetase-2 (*Lars2*) expressing B cells inhibits mouse colon cancer growth (64). *Tgfβ1* expression in B cells is upregulated by Lars2 through metabolite nicotinamide adenine dinucleotide dependent SIRT1 regulation (64). Conditional knockout *Tgfβ1* in B cells reduced tumor growth and correlated with reduced regulatory T cells. Similarly, Li et al. reported that Sting agonist induced regulatory B cells to secrete IL-10 and IL-35 to suppress mouse PDAC growth (65). B cell specific knockout of *Sting*, *Il-10*, or *Il-35* reduced tumor growth. Furthermore, a combination of Sting agonist and IL-35 blocking antibody reduced tumor growth. Shalapour et al. reported that IgA secreting plasma cells suppress immune control of mouse fibrotic liver tumor model through expression of PD-L1 and IL-10 (66). In this model, IgA knockout mice have reduced tumor growth, and the tumor control is dependent on CD8 T cells. This study postulated IL-10 to be a critical B cell-derived immunosuppressive agent; however, its role was not directly proven.

Zhang et al. reported that the neurotransmitter gamma-aminobutyric acid (GABA) derived from B cells may enhance mouse tumor growth (67). The authors observed that GABA synthesis is increased in B cells during immunization or *in vitro* activation. GABA supplementation in B cell deficient µMT mice increased tumor growth, while it had no effect in wild-type mice. Conditional knockout of the GABA synthesis enzyme *Gad1* in B cells also led to reduced tumor growth. Using an *in vitro* cell culture study, GABA induced IL-10 expression in macrophages and inhibited T cell activation, implying a dual function of GABA on both cell types. This study provided an interesting example of how B cell derived GABA may function as an unconventional immune cell modulator in cancer.

One study reported that B cell derived extracellular vesicles (EVs) inhibit CD8 T cell response in mouse tumor models during chemotherapy (68). The authors found that B cell derived EVs expressed the ATPases CD39 and CD73, which inhibited CD8 T cell function *in vitro* through hydrolysis of ATP into immunosuppressive adenosine. Injection of B cell derived EVs reduced transplanted MC38 colon tumor response to cyclophosphamide, which is dependent upon functional CD4 and CD8 T cells. Conditional knockout of the secretion regulator *Rab27a* or *Hif1a* in B cells resulted in a better tumor response to chemotherapy in the B16F10 murine melanoma model and correlated with reduced B cells derived EVs. However, it was not determined if *Rab27a* or *Hif1* deficiency also impacts other secretory processes in B cells, which may confound the conclusion.

It is worth noting that the roles of B cells in the above-cited studies in this section are mostly immunosuppressive, contrasting with the immune activating functions in studies described in sections of antibody effector function and T cell activation. These discrepancies are likely due to the use of different tumor models and their variations in immunogenicity, and will be further discussed below. It is also noteworthy that the antigen specificities of studies in this section are undefined; therefore, the contribution of antigen specific B cell function is unknown.

## Discussion

The recent advances in cancer immunotherapy, together with new technologies such as single cell sequencing, have advanced our understanding of B cells in cancer. The few examples highlighted in this review provide a snapshot of B cell antigen reactivity and their function in cancer.

The currently identified B cell antigens in cancer are broadly separated into viral and non-viral antigens. Virus associated cancers inducing an antiviral B cell response are expected and highlighted with HPV positive HNSCC (38) and EBV positive NPC (41). It is interesting to note that most of the identified viral cancer antigens are intracellular proteins, exemplified by E2, E6, and E7 antigens from HPV positive HNSCC, as well as EBNA1 and VCA antigens in EBV positive NPC. It is known that HPV infection can induce antibodies targeting the surface capsid protein L1, and this B cell response contributes to spontaneous viral clearance and HPV prevention through vaccination (69). In HPV positive HNSCC, the surface antigen L1 is expressed much lower than E2, E6 and E7, the latter being critical for promoting epithelial cell proliferation and viral replication (70). Similarly, though antibody targeting EBV surface glycoprotein antigens (such as gH/gL, gB, gp350) have been reported in natural EBV infections and EBV positive NPC (71), the dominant antibody responses in cancer targets intracellular proteins, such as EBNA1 and VCA (41). This is likely due to EBV in NPC primarily residing in a latent state, with high expression of the oncogenic nuclear antigen EBNA1, and low surface antigens expression (72). B cells likely get access to the intracellular viral antigens likely during the antigen release in cancer cells death, such as necrosis and immunogenic cell death. B cells targeting intracellular viral antigens are unlikely to be directly toxic to EBV infected cancer cells through secreted antibodies. Rather, they may function in the presentation of intracellular viral antigens to CD8 and CD4 T cells, thereby increasing T cell mediated tumor control.

In cancers without an associated viral agent, such as PDAC (57), melanoma (56), and ovarian cancer (51), screening B cell derived antibodies has largely revealed a dominant antibody response to self-antigens. These single B cell approach results generally agree with previous serologic studies, which also identified self-antigens as dominant B cell antigens in cancer (37). This autoreactivity likely results from the chronic inflammation in cancer, leading to a break in immune tolerance. B cells reactive to self-antigens are controlled by both central and peripheral tolerance mechanisms. In central tolerance, bone marrow-born B cells with high-affinity selfreactive BCRs go through clonal deletion, light chain editing or become anergic (6). Despite this, about 20% of naïve B cells have been reported to be self-reactive (7). These low affinity autoreactive B cells are inhibited from becoming pathogenic through peripheral tolerance under normal physiology (8). Peripheral tolerance mechanisms include anergy, antigen competition, receptor editing, apoptosis, and others (73). However, under the chronic inflammatory state, these mechanisms are loosened and can be broken (8). While some of the discussed autoreactive antibodies are germline encoded (51), it is of note that for class switching and SHM to occur, T cell tolerance must also be perturbed. This is exemplified in individuals deficient in CD3 or AIRE, as these patients have heightened B cell autoreactivity (74). It is currently unclear how T cell tolerance is disrupted in human cancers. While loss of self-tolerance can occur for both intracellular and extracellular antigens (75), it is interesting to note that the intracellular localization of autoantigens predisposes to autoantibody production when antigen localization is directly compared (76). The intracellular antigens are likely becoming accessible to B cells in cancer during cancer cell death.

It is noteworthy to mention that most of the identified autoantibody responses in cancer are also reported in autoimmune diseases, such as auaoantibody responses to actin (77, 78), tubulin (79–81) and RUVBL2 (82). These reactions may be associated with the abundance of those antigens, as well as their antigenic potential. Despite the similar formation of autoantibodies, there is no overt evidence that autoimmune disease leads to cancer. For example, while MCB has been reported to have anti-actin autoantibodies (55), and there is no strong documentation of the association with autoimmune disease in clinical multivariate analysis (83). Furthermore, while beta-actin antibody, one of several cytoskeletal antibodies grouped as anti-smooth muscle (SMA) antibody, is often associated with autoimmune hepatitis, however, the risk of cancer development in these patients is more skewed to hepatocellular carcinoma than breast cancer (84). Moreover, melanoma patients are more likely to be associated with autoimmune disease in general (85). Anti-tubulin autoantibodies have been reported in melanoma (56), and anti-tubulin antibodies are often detected in patients typically with autoimmune disease related to neuropsychiatric lupus, chronic inflammatory demyelinating polyneuropathy, and autoimmune liver disease (79–81). Interestingly, the co-existence of melanoma and autoimmune disease correlates with better immunotherapeutic outcomes (85). Conversely, the enhancement of certain autoantibody responses in cancer may lead to paraneoplastic syndromes (86), and checkpoint blockade therapy in cancer can have autoimmune side effects (87, 88).

It is worth noting that most cancer antigen studies described in this review are from immunologically cold cancers, such as PDAC and HGSOC, or melanoma prior to immunotherapy. It is still unknown what the B cell antigen landscape is in more immunogenic cancers, such as melanoma and smoking related lung cancer, and of particular interest, how the landscape changes during response to immunotherapy. With this said, there are some reports that autoantibodies responses are enhanced in immunotherapy (87, 88), so it is possible that these autoreactive B cell responses may also be prevalent in immunogenic tumors.

The function of B cells in cancer has long been contradicted in different tumor model studies, and further understanding of B cell antigen specificity will help clarify those issues. We have categorized the function of B cells into antibody effector function, T cell activation, and immunomodulatory molecule secretion. In the above studies, the authors mostly focused on one category of B cell function, while neglecting others. B cells likely function via multiple parallel mechanisms simultaneously in cancer. In addition, studies on B and T cells are typically done using murine cancer models with ectopic antigens expression or using tumors with carcinogen induced immunogenicity; while studies on the roles of B cell secretion are mostly done in poorly immunogenic models without examination of antigen specificity. These intrinsic immunogenicity differences likely bias the B cell response and its functional assessment. Careful comparison of B cells functions in different immunogenic settings and in the context of antigen recognition will be required to fully address the role of B cells in cancer.

Translational applications of B cell antigens in cancer are still in their early stages. The B cell response in cancer can be used as a diagnostic tool to screen or monitor cancer progression through either serological or histological approaches. Examples are emerging, such as the use of TLS histology as a prognostic factor for cancer immunotherapy (1), or anti-EBV serological response for screening of NPC (43). However, the applicability of using identified B cell antibodies/antigens for cancer therapeutics is limited. Most of the identified B cell specific viral antigens are intracellular proteins, such as HPV E2 and EBV EBNA1, which limit direct targeting in cancer. Ongoing studies also aim to use viral vaccines to induce both B and T cells for cancer control (89, 90).

The non-viral antigens identified so far are mostly self-antigens, raising the question of whether B cells targeting these autoantigens can affect tumor outcomes. B cells targeting surface self-antigens can potentially lead to tumor targeting through the aforementioned antibody effector functions; however, this remains to be demonstrated in a human cancer setting. Relatedly, the enhancement of these self-responses may lead to autoimmune diseases in cancer, and is manifested in examples of paraneoplastic autoimmunity (86). Instead, for the majority of intracellular autoantigens, antibodies from B cells cannot exert a direct effector function on viable tumor cells. However, B cells expressing an autoreactive BCR may instead endocytose intracellular autoantigens released during tumor cell death, promoting anti-tumor immunity through antigen presentation and cytokine expression. While human cancer immunotherapy has been reported to induce autoimmunity, including increased autoantibody response (87, 88), this autoimmunity also positively correlates with immunotherapy efficacy (91). It will be important to elucidate whether B cells contribute to tumor control, or just correlate with it, in the context of immunotherapy.

Facilitated by accumulating studies on comprehensive profiling of the BCR in cancer (12–14), it is foreseeable that the identification of B cell antigens in cancer will also increase. Based on available studies, intracellular autoantigens will likely constitute the major B cell antigens in cancer. Thus, future studies will likely require a deeper screening of B cell repertoire with low frequency clones to identify novel cancer specific surface antigens. In addition, it will be reasonable to speculate that cancer specific B cell antigens may be more prevalent in immunogenic cancers, especially during immunotherapy.

In all, we are cautiously optimistic that the advancements in the understanding of the B cell antigen reactivity in cancer will lead to a better understanding of the B cell’s role in cancer, and provide potential therapeutic targets and diagnostic tools. The field is poised on the verge of diving deeper into the B cells antigen discovery with great translational potential.
